# Duration of Bloodless Operations

**Published:** 1875-10

**Authors:** 


Duration of Bloodless Operations.—At a recent meeting of the fourth Congress of the German Surgical Society, Professor

Langenbeck stated his opinion that it was of extreme importance to determine more accurately than has as yet been done how long a limb can be deprived of blood, during a bloodless operation, without danger either to it or to the patient. He thought that the constriction could be kept up for a very long time without fear of gangrene, if the patient could only overcome the disagreeable sensation which the bandage caused. We, however, particularly need experiments on animals, to determine the exact limit of safety. In long operations on the bones—for example, resections—we can see that the bloodlessness is not complete in them as in the soft parts around, and that capillary bleeding occurs from them when not a drop of blood issues from the muscles. Prof. Langenbeck continued: "I have recently kept up the constriction in excisions of the ankle joint for an hour and a half, without any ill effects. After this operation I always find a plaster-of-Paris bandage the most comfortable and useful dressing; but till lately there was one great disadvantage attending its use—namely, that it became soaked with blood as soon as it was put on. In the last two cases in which I have operated, I have kept up the compression until the bandage was perfectly hard, and then cut large openings in it so that the whole wound could be seen, and only then removed the constriction. The abundant hemorrhage which now occurs from the vessels of the periosteum and the bone soon stops, if the femoral artery is compress for a short time; with a little care the blood can thus be kept from the soaking into the bandage,"—*Med. Times and Gaz., July* 31, 1875.—*Monthly Abstract.*

